# The Annual Museum

**Published:** 1894-08-25

**Authors:** 


### THE BRITISH MEDICAL ASSOCIATION AT BRISTOL.

### THE ANNUAL MUSEUM.

(2ND NOTICE.)

A LARGE display of attractive character close to the main entrance was to be seen on the stall of JOHN RICHARDSON AND CO., Leicester. The enormous variety of pills, tablets, soaps, and capsules, for which this house is famous, were formed into an exhibit of great beauty and flanked by one of their handsome walnut dispensing cabinets filled with bottles and counter. We can only mention their sol. ammon aromat, an inexpensive substitute for sp. ammon. aromat, the bichromate of potash capsules for gastritis, and their effervescent preparations. Their collection of surgical instruments contained Ehrhardts' Powder Insufflator, which discharges the exact amount desired, and many improved laryngeal, uterine, and other articles.

THE NIBESTOS FILTERS COMPANY show various types of their filters, large and small, which drew much attention. There is no attempt in these filters at purifying a clogged and worn out filtering medium. The cleansing is carried out easily by removing, and replacing the soiled layer by a new one as often as desired at a trifling cost. Moreover, they can be regulated for different degrees of pressure. Thus they seem to overcome the chief deficiencies in ordinary filters.

The Petroleum Emulsion of the ANGIER CHEMICAL COMPANY was brought forward on another stall. It is palatable and easily borne by the stomach, and is said not only to be a capital vehicle for the hypophosphates of lime and soda which it contains, but to have an antiseptic and soothing effect on the gastric intestinal tract. Hence it is recommended both for pulmonary affections and also for catarrhal states of the digestive organs.

The soaps of CHRISTOPHER THOMAS AND BROS. are one of the most important manufactures of Bristol. Their Carbolic Soap is guaranteed to contain 5 per cent. of Calvert's white crystallised acid. Two of their articles are worth special notice, their Olive-oil Soap, which has been highly recommended for over a century both as a toilet article and for use in skin troubles, and the Assyrian Toilet Soap, which is of exquisite perfume and high character. They contain no free alkali and yet are not superfatted.

A remarkable Obstetric Binder Grip has been devised by MRS. HUNTER, of Charlotte Street, Bristol, which does away with pins and straps, while any ordinary material forms with it a smooth, perfectly-fitting binder.

MESSRS. HORLICK AND CO.'S Malted Milk is a sterilized milk food free from starch and cane sugar. The ease with which it is prepared was shown on the stall, mere solution in water at the feeding temperature suffices to produce a perfectly-cooked food. This, of course, as they point out, means a great saving of labour for nurses, and the preparation keeps well in any climate.

THE FRAME FOOD COMPANY showed their frame food extract diet, jelly, and tabloids. The latter we have tried and find them to be very palatable. The jelly can be eaten as a jam on bread, and for all their products a high nutritive value is claimed, since over 10 per cent. of phosphates are said to be found in the extract.

PROFESSOR SNEDEKOR'S Thermogen is a device for generating heat and maintaining a constant temperature for any length of time from 80 to 200 deg. It can be used to replace hot bottles or to maintain a fomentation at the original temperature. One form is interwoven in a quilt to keep up the warmth of the patient on the operating table, and another is designed to form a Turkish bath in the house. The invention appears most valuable and meets a long-felt want. Essentially it consists of a flexible electrical resistance coil embedded in a fire and water proof insulator which can be worked from lighting currents or from accumulators.

KING, MENDHAM, AND CO., Bristol, showed their Wimshurst Static Electrical Machines and their special Sulphate of Mercury Battery designed for use in Apostoli's treatment of fibroids. This battery can also be employed for a surgical. Their lamp motors for dental drills, portable batteries, accumulators, and galvanometers are too numerous to mention, but display great ingenuity and good workmanship.

THE CANADIAN CLUB WHISKEY fully maintained its popularity, though it would appear that independent analysis was perpetually required, in spite of the high recommendations it has received from the medical journals. The flavour is good, and the purity and age of the spirit appears to be well guaranteed.

B. Kuhn, of 36, St. Mary-at-Hill, London, among numerous other preparations gave a prominent place to Finkler's Papaine. This much-discussed preparation was illustrated by photos of the papaya tree, and many samples of the products. The Ethyl Choride Spray of Dr. Bengué is an invention already found of great use by many practitioners. The local anæsthesia which it produces is so rapid, reliable and harmless, and the spray so handy and simple that it must become almost a necessity. By his Anestile anæsthesia is produced even more quickly and over a larger surface.

The Peat Industries Syndicate showed some wonderful specimens of the beautifully manufactured material they sell. It is almost incredible that the fine silky fibres which they form into dressings, mattresses, socks, &c., can be derived from ordinary peat. The power of absorption possessed by this material, together with its power of preventing decomposition, would suffice to make it a valuable dressing for surgeons, even if its cheapness and softness were not so marked. The stall was altogether a most interesting one, and the mattresses and dressings should command a large sale.

Mayer and Meltzer showed several novelties. Coward's Saccharometer enables the quantitative estimate of sugar to be made in a few minutes at the bedside or in the consulting room. The estimation is made by a graduated tube. The little case containing it also holds Doremus ureameter and Esbach's tube for calculating albumen. We should also refer to Whitmore's dressing tray with a central drainage tube to prevent wetting the bedding. Their Aseptic Cases for the carriage of instruments and apparatus for operations, and their portable Faradic batteries are remarkably ingenious. The battery contains a sealed cell intended to last for months without renewal. The novelties among their aural laryngeal and nasal instruments included Lennox Brown's Tonsil bistoury and scraper, a new écraseur by Macdonald, and other highly interesting contrivances.

When so much discussion is taking place on the comparative safety of anæsthetics it is almost needless to direct attention to the efforts which are being made to manufacture thoroughly reliable articles.

Salamon and Co. had on view samples of their purest chloroform and absolute ether. Pleasant to inhale, they have been very strongly recommended by the medical journals for their high degree of purity.

The collections of the products of English spas showed the healthy activity with which native waters are being brought forward and are gaining the popularity they deserve.

The Woodall Spa exhibited their powerful Bromo-Iodine Water and several extracts and preparations from it.

The Leamington Town Improvement Association had specimens of water, and gave away a charming guide book full of beautiful views of the town and the neighbourhood. The views are some of the best we have seen, and will surprise many who have neglected the lovely scenery and antiquities of this county. With such a group of places of surpassing interest close to this rising health resort there is no wonder that Americans make it often their chief place of stay in England. Great improvements have been carried out with energy and success in attracting visitors to these "unrivalled" waters.

The Harrogate Corporation sent a lovely collection of photos of their town and samples of the waters which are valuable substitutes for Kissingen and other Continental spas. There is an astonishing variety of waters, no less than 80 springs being known. In the handbook to the town which was distributed, analyses of the 14 principal ones are given, together with an account of the new baths and the interesting spots in the neighbourhood.

We must not forget the exhibit of the Oliver Urinary Test papers for sugar, albumen, bile, &c., shown by Messrs. Wilson and Son, of Harrogate. Their little pocket case of these tests for 12s. 6d. is of the greatest utility to the busy paactitioner. Clifton has now an opportunity of entering the lists with these well-known spas through the energy of Mr. Newnes, who opened during the exhibition week the splendid new spa on the rocks above the old Hotwells.

(*To be continued.*)

